# Reduced cervical cancer incidence and mortality in Canada: national data from 1932 to 2006

**DOI:** 10.1186/1471-2458-12-992

**Published:** 2012-11-16

**Authors:** James A Dickinson, Agata Stankiewicz, Cathy Popadiuk, Lisa Pogany, Jay Onysko, Anthony B Miller

**Affiliations:** 1 Departments of Family Medicine and Community Health Sciences, University of Calgary, Calgary, Alberta, Canada; 2 Public Health Agency of Canada, Ottawa, Ontario, Canada; 3 Department of Women’s Health, Memorial University, St John’s, Newfoundland, Canada; 4 Dalla Lana School of Public Health, University of Toronto, Toronto, Ontario, Canada; 5 Family Medicine and Primary Care Research Office, University of Calgary G012, Health Sciences Centre, 3330 Hospital Drive NW, Calgary, Alberta, T2N 4N1, Canada

**Keywords:** Cervical cancer, Epidemiology, Incidence, Mortality, Trends, Screening, Canada

## Abstract

**Background:**

High levels of participation in cervical screening are reported in Canada from the 1970’s as a result of early uptake of the Pap smear and universal Medicare. Despite recommendations to the contrary, the programs have featured early age of initiation of screening and frequent screening intervals. Other countries have achieved successful outcomes without such features. We analyzed national data to better understand mortality and incidence trends, and their relationships to screening.

**Methods:**

The Canadian Cancer Registry, National Cancer Incidence Reporting System, and the Canadian Vital Statistics Database were used to measure mortality and incidence rates. Cases and deaths from invasive cervical cancer were classified by 5 year age groups at diagnosis and death (15 to 19 years through to 80 to 84 years), year of diagnosis (1972 to 2006), and year of death (1932 to 2006). Probabilities of developing and dying from cervical cancer were calculated for age-specific mortality and incidence. The proportion of women reporting a timely Pap test was estimated for 1978 to 2006.

**Results:**

Cervical cancer mortality has declined steadily from a peak of 13.5 to 2.2 per 100,000 (83%,) between 1952 and 2006, and 71% between 1972 and 2006. Incidence of invasive cervical cancer has declined by 58% since 1972. These declines have occurred more among older age groups than younger. Invasive cervical cancer incidence and mortality is less in each successive birth cohort of women. Participation rates in screening are high especially in women under age 50.

**Conclusions:**

Despite increasing risk factors for cervical cancer, both incidence and mortality have declined over time, across age groups, and across birth cohorts. Earlier increasing mortality (1932 – 1950) was likely related to improved classification of cancers and the early subsequent reduction (1950 – 1970) to improved treatment. Recent improvements in incidence and mortality are likely due to high rates of screening. For women under age 30 years there are low rates of disease but lesser improvement related to screening.

## Background

Cervical cancer is one of the most common cancers among women worldwide: third in incidence overall [1]. In Canada, cervical cancer was frequent, but now ranks 11^th^ for incidence and 16^th^ for cancer-related mortality [1-3]. Approximately 1,300 women were diagnosed and about 350 women died from the disease in Canada during 2011 [4,5].

Canada was an early adopter of cervical cancer screening from 1949 in British Columbia with gradual intensification across the country [6]. Beginning in the 1960’s, intensification of screening was linked to oral contraceptive prescriptions and pre- and post-natal care resulting in high screening rates among women younger than 35 [6,7]. In the early 1970’s cervical cancer screening became partially subsidized and ultimately free of charge in 1984. Thereafter, uptake of screening increased [8]. By 1973, the screening rate was near 50%, and by 1997, over 75% among women between 18 and 64 years [7]. During this time period, annual screening was the norm, beginning at age 18 or even younger and ceasing at age 69 [9,10]. By 1976, screening was linked to a reduction in mortality from cancer of the uterus as a whole, which includes cancer of the cervix [11]. Screening was adopted without a randomized trial to demonstrate its efficacy, and there is limited data to elucidate which ages or subgroups of women should be screened [12]. The need for screening at ages younger than 25 has been questioned because of the low incidence and high rates of false positives, causing large numbers of referrals for colposcopy [13].

The understanding that nearly all cervical cancer is caused by oncogenic strains of the human papillomavirus (HPV) has assisted in better interpretation of the associations between behavior and cervical cancer. The United Kingdom has observed fluctuations of cervical cancer incidence: women who reached early adulthood in times of war had higher rates than those who matured during peace [14,15]. Other European countries have demonstrated changes in incidence and mortality because of period and cohort effects related to the coverage and effectiveness of screening policies [16,17].

Canada has near universal availability of cervical screening, and has had policies encouraging starting screening early in life and repeating frequently. There is also high quality long-term national data on mortality and incidence. To assess the effect of this intense screening, we used national data on mortality and incidence to analyze changes in the mortality and incidence of invasive cervical cancer over time, by age group and birth cohort, and relate this to screening activity and other factors that have affected this cancer.

## Methods

### Data sources

Mortality data were obtained from the Canadian Vital Statistics Database (1950 – 2006) and annual Statistics Canada publications (1932–1949) [18,19]. Incidence data were obtained from the Canadian Cancer Registry (1992 – 2006) and the National Cancer Incidence Reporting System (1972 – 1991) [4,19,20]. Multiple primary coding rules of the International Agency for Research on Cancer were utilized [21]. For incidence, cancers (C53) were classified according to the International Classification of Disease for Oncology Third Edition. For mortality, cancers (C53) were classified according to the International Statistical Classification of Diseases and Related Health Problems, Tenth Revision [22,23]. Deaths were included when cervical cancer was determined to be the underlying cause of death. Population estimates were obtained from Statistics Canada [18,24-42] and are based on intercensal estimates from 1932 – 2005 and post censal estimates for 2006 and 2007. Estimates from 1932 to 1970 were not adjusted for enumeration undercounts resulting in small relative drops in incidence for the years from 1971. For 1971 the ratio of the adjusted estimate to the unadjusted census population estimate for women is 1.015. Direct age standardization was performed using the Canadian 1991 population as standard.

Ethical approval was not required, since routinely collected de-identified data were analysed within the confidential environment of the Public Health Agency of Canada by their staff (JO, AS, LP), and only aggregate data were released, following standard rules for suppression of cells containing very small numbers.

### Analysis

Data were classified by 5 year age groups at diagnosis and death (15 to 19 years through to 80 to 84 years), year of diagnosis (1972 to 2006) and year of death (1932 to 2006). The 80 to 84 age group was chosen as the cut-off because it was the eldest available 5-year age group. Incidence and mortality rates for each category were calculated by dividing the number of cases or deaths in each category by the census population. Percent reductions among 5-year age groups were calculated using five-year period mortality and incidence rates. Denominators were not adjusted for hysterectomy. Rates based on 1 to 5 observations for incidence and 1 to 4 observations for mortality were suppressed for confidentiality purposes

Probabilities of developing and dying from cervical cancer were calculated for age-specific mortality (1952, 1972 and 2006) and incidence (1972 and 2006). Respective life tables were based on 1951 to 1953, 1971 to 1973 and 2005 to 2007 all-cause mortality rates. Calculation of the probability of developing and dying from cancer were based on standard methods [43,44].

Data used to estimate the proportion of women with a timely Pap test uptake were obtained from the Canada Health Survey 1978–1979, Health Promotion Survey 1985, 1990, National Population Health Survey 1994–1995, 1996–1997, 1998–1999, and Canadian Community Health Survey cycles 1.1 (2001), 2.1 (2003), 3.1 (2005), and Canadian Community Health Survey - Annual Component, 2008. Timely cervical cancer screening was defined as having had a pap smear test within 3 years for all surveys except the CHS 1978–79 which used a two year period. Denominators for these survey reports were adjusted for hysterectomy from 1996–1997 onwards. Detailed information on the above survey designs, sample sizes and methodologies is available upon request.

## Results

### Overall

**Figure 1:**
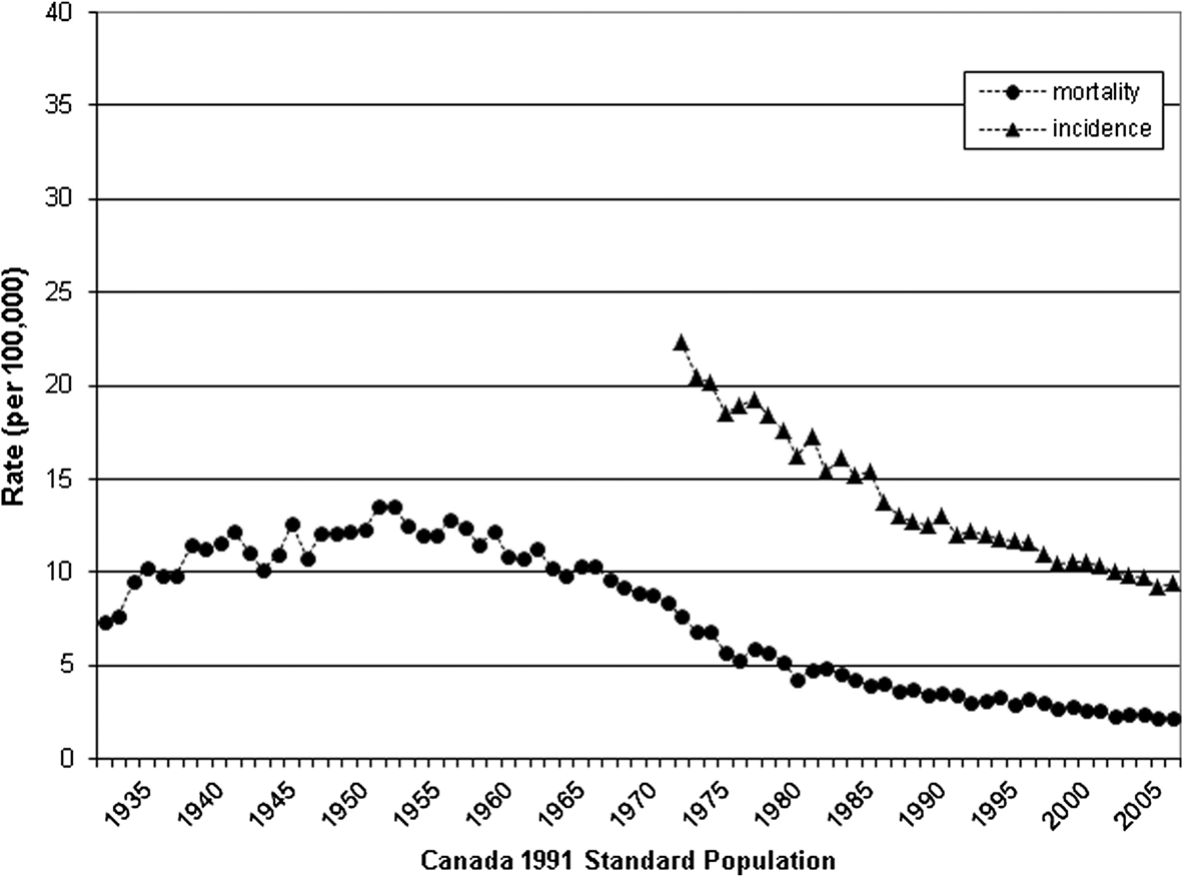
Age standardized mortality and incidence of cervical cancer in Canada.

**Figure 2:**
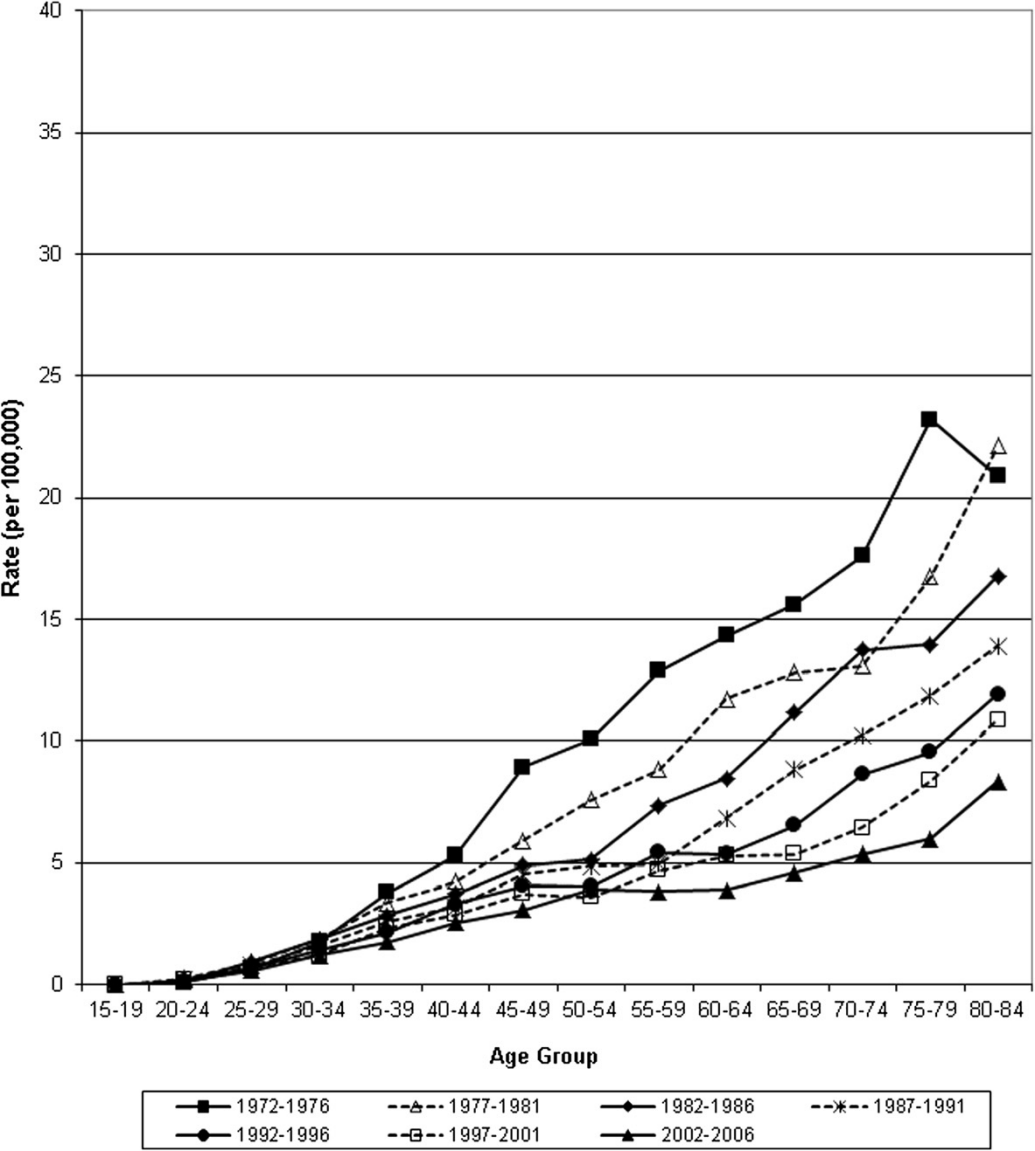
Age-specific mortality from invasive cervical cancer in Canada, 1972–2006.

**Figure 3:**
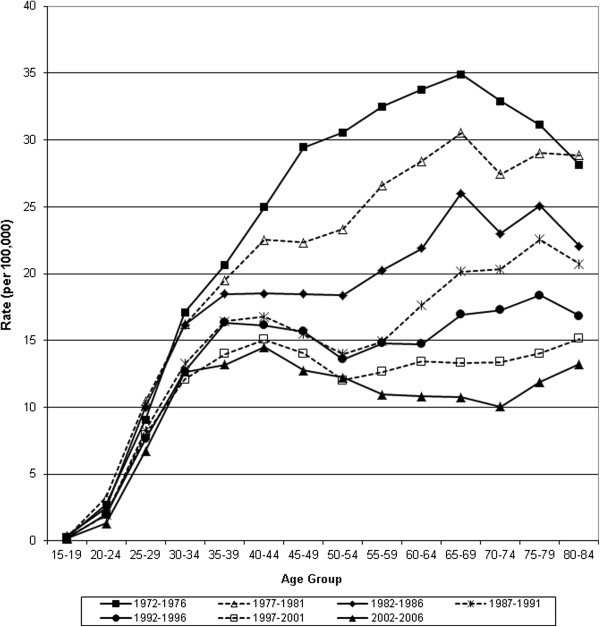
Age-specific incidence of invasive cervical cancer for 5 year periods, 1972–2006.

Age standardized mortality from invasive cervical cancer declined 83% from 1952 to 2006 (13.2 to 2.2 per 100,000 women respectively). About half of the decline occurred between 1972 and 2006 (7.7 to 2.2 per 100,000 women respectively) (Table 1 & Figure 1). Age-standardized incidence declined 58% between 1972 (22.3 per 100,000) and 2006 (9.4 per 100,000) (Figure 1 & Table 2). This was notable over the age of 40, with lesser effects below that age, except for a relative reduction of 50% for women aged 20–24. Figures 2 and 3 show a steady sequential reduction of age-specific mortality and incidence from 1972 to 2006. The greatest declines in both mortality and incidence are observed in age groups over 45 years with reductions as high as 74% in mortality and 69% in incidence (Tables 1 &2).

**Table 1:** Reduction in age specific mortality rates from invasive cervical cancer in Canada: 1952 – 2006 and 1972 – 2006

**Age Group**	**1952 – 1956**		**1972 – 1976**		**2002 – 2006**		**1952 – 2006**	**1972 – 2006**
	**Deaths**	**Rate (Per 100,000)**	**Deaths**	**Rate (Per 100,000)**	**Deaths**	**Rate (Per 100,000)**	**% Mortality Rate Reduction**	**% Mortality Rate Reduction**
15 to 19	0	0	*	*	0	0.0	--*	--*
20 to 24	9	0.3	5	0.1	9	0.2	--*	--*
25 to 29	51	1.7	30	0.6	31	0.6	--*	--*
30 to 34	137	4.7	66	1.8	65	1.2	75	32
35 to 39	223	8.3	121	3.8	105	1.7	79	54
40 to 44	337	14.3	167	5.3	172	2.5	82	52
45 to 49	417	21.0	280	8.9	197	3.1	85	66
50 to 54	357	21.0	303	10.1	223	3.9	81	61
55 to 59	325	21.9	326	12.9	186	3.8	82	70
60 to 64	357	28.2	319	14.3	145	3.9	86	73
65 to 69	311	28.5	281	15.6	141	4.6	84	70
70 to 74	238	27.7	244	17.6	149	5.4	81	70
75 to 79	176	32.9	234	23.2	145	6.0	82	74
80 to 84	81	28.3	135	20.9	155	8.3	71	60

*Deaths, mortality rate and mortality reduction is not provided due to small numbers of cases and the resulting susceptibility to spurious variation.

**Table 2:** Reduction in age specific incidence rates from invasive cervical cancer in Canada: 1952 – 2006 and 1972 – 2006

**Age Group**	**1972 – 1976**		**2002 – 2006**		**% Incidence Rate Reduction**
	**New Cases**	**Rate (per 100,000) 1972 to 1976**	**New Cases**	**Rate (per 100,000) 2002 to 2006**	
15 to 19	15	0.3	9	0.2	35
20 to 24	143	2.7	70	1.3	52
25 to 29	429	9.1	355	6.7	26
30 to 34	643	17.1	689	12.7	26
35 to 39	660	20.7	794	13.2	36
40 to 44	787	25.0	982	14.5	42
45 to 49	924	29.4	821	12.8	57
50 to 54	919	30.6	694	12.2	60
55 to 59	822	32.5	532	10.9	66
60 to 64	752	33.8	404	10.8	68
65 to 69	629	34.9	329	10.8	69
70 to 74	456	32.9	279	10.0	69
75 to 79	314	31.1	288	11.9	62
80 to 84	182	28.2	246	13.2	53

### Trends by calendar year

**Figure 4:**
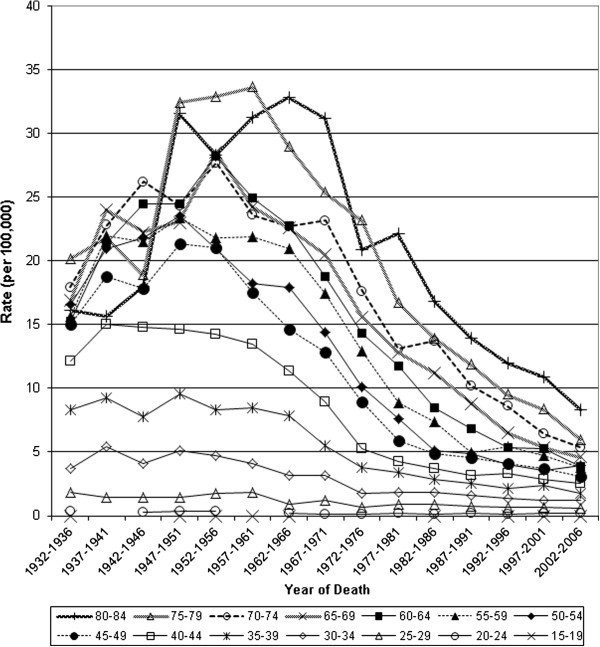
Mortality from invasive cervical cancer in Canada, 1932–2006: trends by year of death.

**Figure 5:**
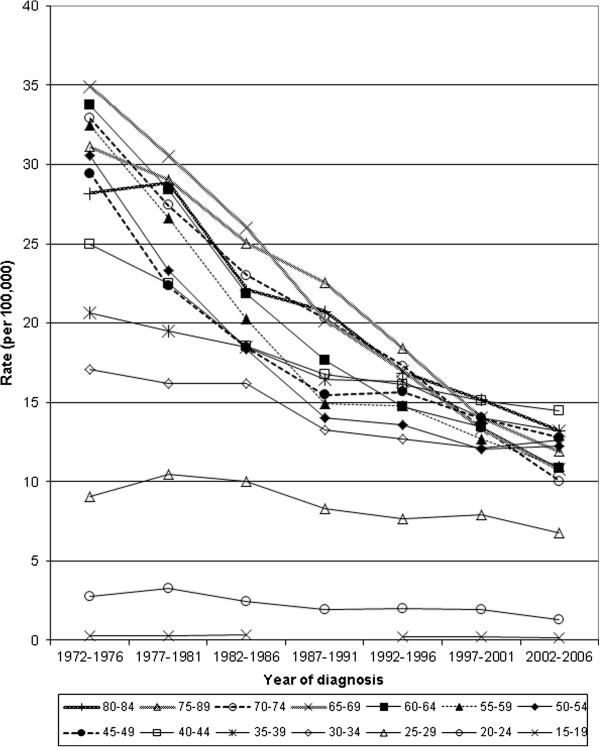
Incidence of invasive cervical cancer in Canada, 1972–2006: trends by year of diagnosis.

After initial rises in mortality for older women, there was a steady decline among women younger than 60 years from the 1940s, and among women over 60 years of age from the 1950 – 1960s (Figure 4). Incidence declined for all age groups from 1972 to 2006, most apparent in women above age 30 and the reduction was larger at ages over 45 years (Figure 5).

### Trends by birth cohort

**Figure 6:**
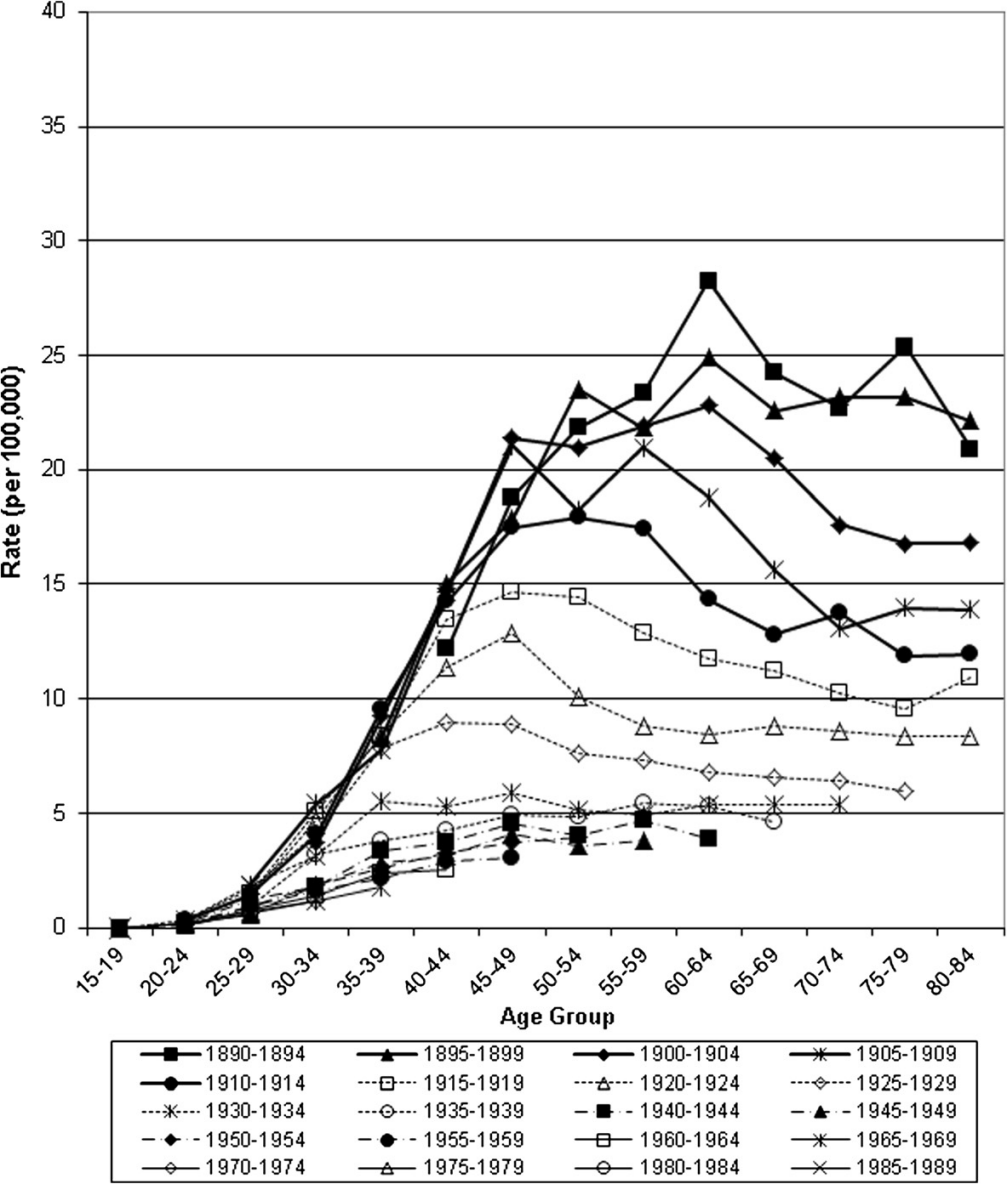
Mortality from invasive cervical cancer in Canada, 1932–2006: trends by year of birth.

**Figure 7:**
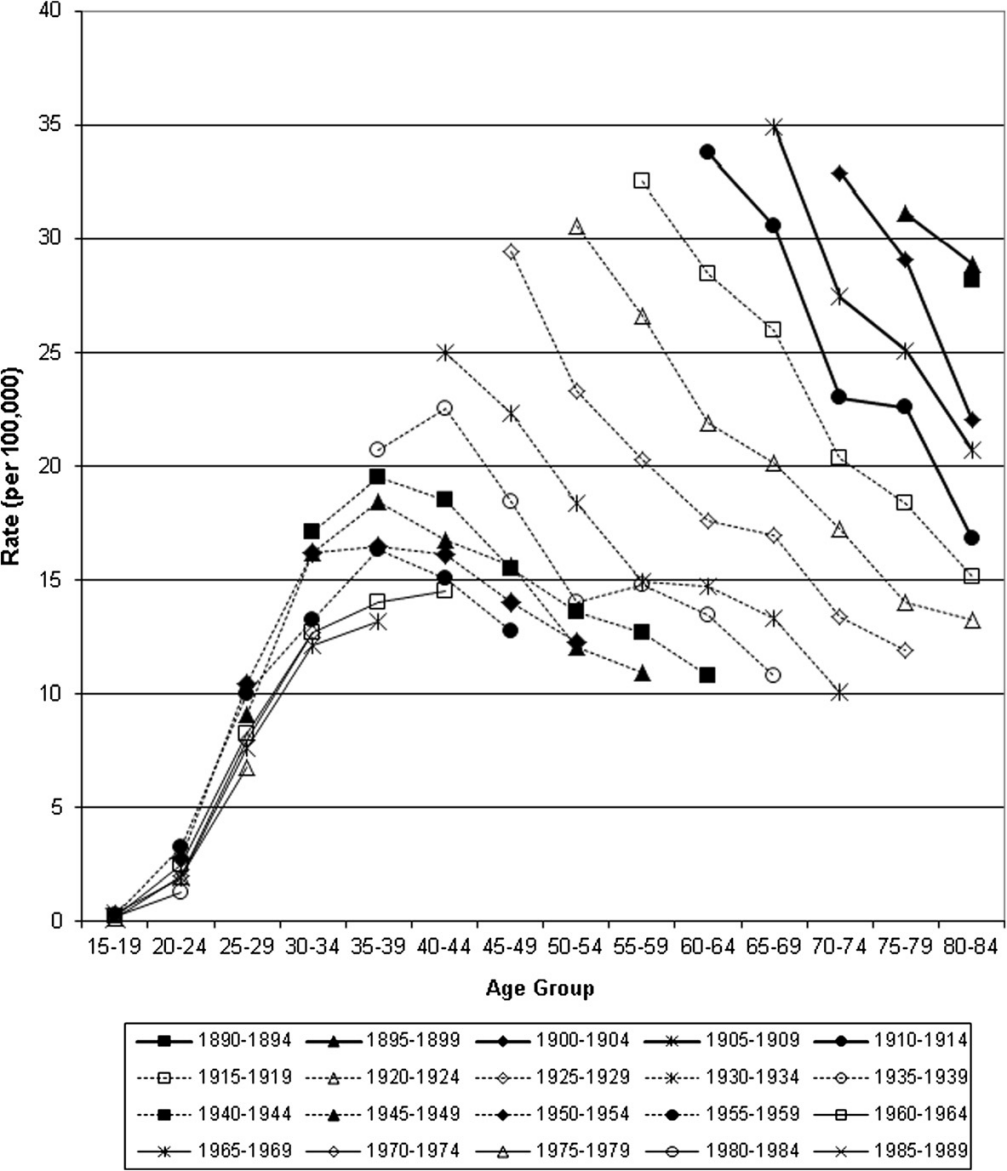
Incidence of invasive cervical cancer in Canada, 1972–2006: trends by year of birth.

Age-specific mortality and incidence were compared by birth cohorts for women born between 1890 and 1989 (Figures 6 &7). A steady progression to lower mortality from the earliest to the most recent birth cohorts is observed with peak mortality dropping from 28.2 deaths to 4.1 deaths per 100,000 women. Peak mortality rates occurred at progressively younger ages from the 1890–94 cohort to 1930–34 cohort. The most recent birth cohorts (1940 to 1989), have lower initial increases in mortality but rates continue to rise with age and it is not possible to discern whether they will drop after the 4^th^ decade. Women among the earliest birth cohorts experienced twice the peak incidence of invasive cervical cancer than women in the birth cohorts after 1950 (Figure 7). In 1952, the lifetime probability of death from cervical cancer (percent) was 0.94, by 1972 it was 0.66, and by 2006, 0.22. The lifetime probability of developing invasive cervical cancer fell from 1.54 to 0.66 (1972 and 2006 respectively).

### Uptake of screening

Canada-wide community health surveys show consistent rates of cervical screening between 1978 and 2006: over 40% among 18–19 year-olds, rising to mostly over 80% during the young adult ages, then dropping thereafter, especially after age 70 (Table 3). The rates are lower in the 1978–9 survey, largely accounted for by the two-year definition of timely uptake.

**Table 3:** Self-reported timely* uptake of Pap test among women aged 18 to 84 years, Canada: 1978/9 to 2008

**Age Group**	**1978/1979 % (95%CI)**	**1985 % (95%CI)**	**1990 % (95%CI)**	**1994/1995 % (95%CI)**	1996/1997** % (95%CI)	1998/1999** % (95%CI)	2001** % (95%CI)	2003** % (95%CI)	2005** % (95%CI)	2008** % (95%CI)
**18-19**	43.2 (35.6 - 51.2)	49.3 (41.6 - 57.0)	54.2 (43.4 - 65.1)	48.5 (40.2 - 56.8)	49.6 (43.0 - 56.1)	44.0 (33.5 - 54.5)	43.8 (40.8 - 46.7)	47.3 (44.0 - 50.6)	42.4 (39.1 - 45.6)	39.8 (35.0 - 44.7)
**20-24**	77.3 (74.1 - 80.2)	83.4 (80.8 - 86.1)	75.4 (70.1 - 80.6)	71.1 (65.2 - 77.0)	72.8 (68.6 - 77.0)	72.5 (67.3 - 77.7)	65.9 (63.7 - 68.1)	70.4 (67.7 - 73.1)	70.0 (67.6 - 72.3)	73.9 (70.7- 77.3)
**25-29**	86.8 (84.9 - 88.4)	90.9 (88.0 - 93.8)	85.5 (82.6 - 88.4)	83.8 (80.1 - 87.5)	85.5 (82.5 - 88.4)	86.8 (82.8 - 90.8)	80.4 (78.5 - 82.2)	81.3 (79.3 - 83.2)	82.5 (80.7 - 84.2)	81.3 (78.6 - 84.1)
**30-34**	79.7 (75.0 - 83.7)	90.9 (88.0 - 93.8)	84.5 (79.5 - 89.6)	83.7 (80.5 - 86.9)	87.3 (85.2 - 89.4)	87.1 (83.9 - 90.4)	81.7 (80.0 - 83.4)	84.6 (83.0 - 86.2)	85.0 (83.6 - 86.4)	83.1 (80.6 - 85.6)
**35-39**	74.3 (70.1 - 78.1)	83.6 (80.6 - 86.6)	82.5 (76.7 - 88.3)	80.9 (76.8 - 85.0)	84.5 (82.1 - 86.8)	85.7 (82.5 - 88.9)	80.5 (78.8 - 82.2)	82.2 (80.5 - 84.0)	82.0 (80.3 - 83.6)	83.2 (80.6 - 85.7)
**40-44**	67.8 (63.0 - 72.2)	83.5 (80.5 - 86.5)	81.3 (75.6 - 87.0)	79.2 (74.6 - 83.0)	81.5 (78.4 - 84.6)	82.9 (78.7 - 87.2)	80.3 (78.8 - 81.7)	82.8 (81.0 - 84.6)	81.2 (79.5 - 82.8)	81.9 (78.8 - 84.9)
**45-49**	70.1 (64.6 - 75.1)	79.2 (72.9 - 85.5)	77.8 (71.3 - 84.4)	75.4 (70.8 - 79.9)	79.6 (76.2 - 83.1)	83.9 (79.3 - 88.6)	78.6 (76.7 - 80.5)	81.3 (79.5 - 83.0)	78.6 (76.6 - 80.7)	82.3 (79.4 - 85.1)
**50-54**	63.8 (56.5 - 70.4)	74.9 (69.2 - 80.6)	75.2 (68.4 - 82.0)	73.5 (68.0 - 79.1)	80.9 (78.0 - 83.7)	85.2 (81.2 - 89.2)	79.8 (77.7 - 81.9)	80.8 (79.1 - 82.5)	79.7 (77.8 - 81.6)	77.4 (73.8 - 81.0)
**55-59**	57.3 (51.3 - 63.1)	69.0 (62.7 - 75.4)	69.3 (62.8 - 75.8)	62.3 (55.5 - 69.1)	72.5 (68.1 - 77.0)	80.5 (74.9 - 86.1)	77.4 (74.9 - 79.8)	81.9 (80.3 - 83.5)	77.6 (75.8 - 79.4)	76.9 (73.3 - 80.5)
**60-64**	42.1 (36.1 - 48.3)	69.7 (64.1 - 75.2)	65.1 (58.5 - 71.8)	60.9 (54.1 - 67.7)	64.8 (59.8 - 69.8)	74.1 (67.5 - 80.7)	72.8 (69.6 - 76.1)	74.7 (72.6 - 76.8)	75.0 (73.1 - 76.9)	71.5 (67.9 - 75.1)
**65-69**	41.3 (35.6 - 47.1)	59.8 (52.1 - 67.4)	55.1 (47.3 - 62.9)	56.4 (50.4 - 62.4)	55.9 (50.4 - 61.4)	61.8 (54.5 - 69.1)	64.8 (61.8 - 67.8)	65.0 (62.6 - 67.4)	63.0 (60.5 - 65.4)	66.8 (62.9 - 70.8)
**70-74**	28.3 (21.8 - 35.8)	50.8 (42.9 - 58.8)	41.1 (32.0 - 50.1)	35.7 (29.9 - 41.5)	46.1 (41.3 - 50.8)	55.4 (46.8 - 64.0)	50.8 (47.6 - 54.0)	54.0 (51.4 - 56.6)	51.6 (49.3 - 53.9)	48.7 (43.8 - 53.5)
**75-79**	19.6 (14.1 - 26.6)	43.5 (33.0 - 54.0)	33.0 (23.6 - 42.5)	31.3 (25.2 - 37.4)	33.7 (28.0 - 39.5)	40.2 (31.1 - 49.3)	37.7 (34.4 - 41.1)	38.1 (35.1 - 41.1)	35.7 (32.9 - 38.4)	27.6 (22.7 - 32.4)
**80-84**	15.2 (9.4 - 23.5)	43.4 (30.9 - 55.9)	35.0 (21.8 - 48.2)	27.6 (18.4 - 36.8)	26.1 (21.0 - 31.2)	26.9 (16.4 - 37.3)	23.9 (20.8 - 27.1)	23.7 (21.0 - 26.5)	23.3 (20.5 - 26.0)	21.4 (17.2 - 25.7)

Timely uptake is defined as within the last 2 years (1978/79) or within the last 3 years (1985 – 2008).

** Hysterectomy corrections made from 1996–7 onwards.

## Discussion

Incidence and mortality from invasive cervical cancer has declined in Canada since the 1950s. For most age groups mortality has declined by over 80% since the 1950s and 50% since the 1970s respectively (Table 1) while incidence declined by more than 50% since the 1970s (Table 2). Mortality reductions are small for women under the age of 30 and greater for older women, with the largest reductions over the age of 50 years. Period analysis suggests rising mortality for older age groups until the 1950s and 1960s, then steady reductions for all age groups from the 1970s. Birth cohort analysis demonstrates differing trajectories between the most recent and distant cohorts: among recent cohorts small increases in mortality rates with age peak at approximately 4 deaths per 100,000 women, compared to over 24 deaths per 100,000 women among those born before 1900. Cohort analysis of incidence demonstrates steady reductions for all age groups, especially over age 40, and extrapolating backwards suggests that Canada previously had a substantially higher incidence.

**Figure 8:**
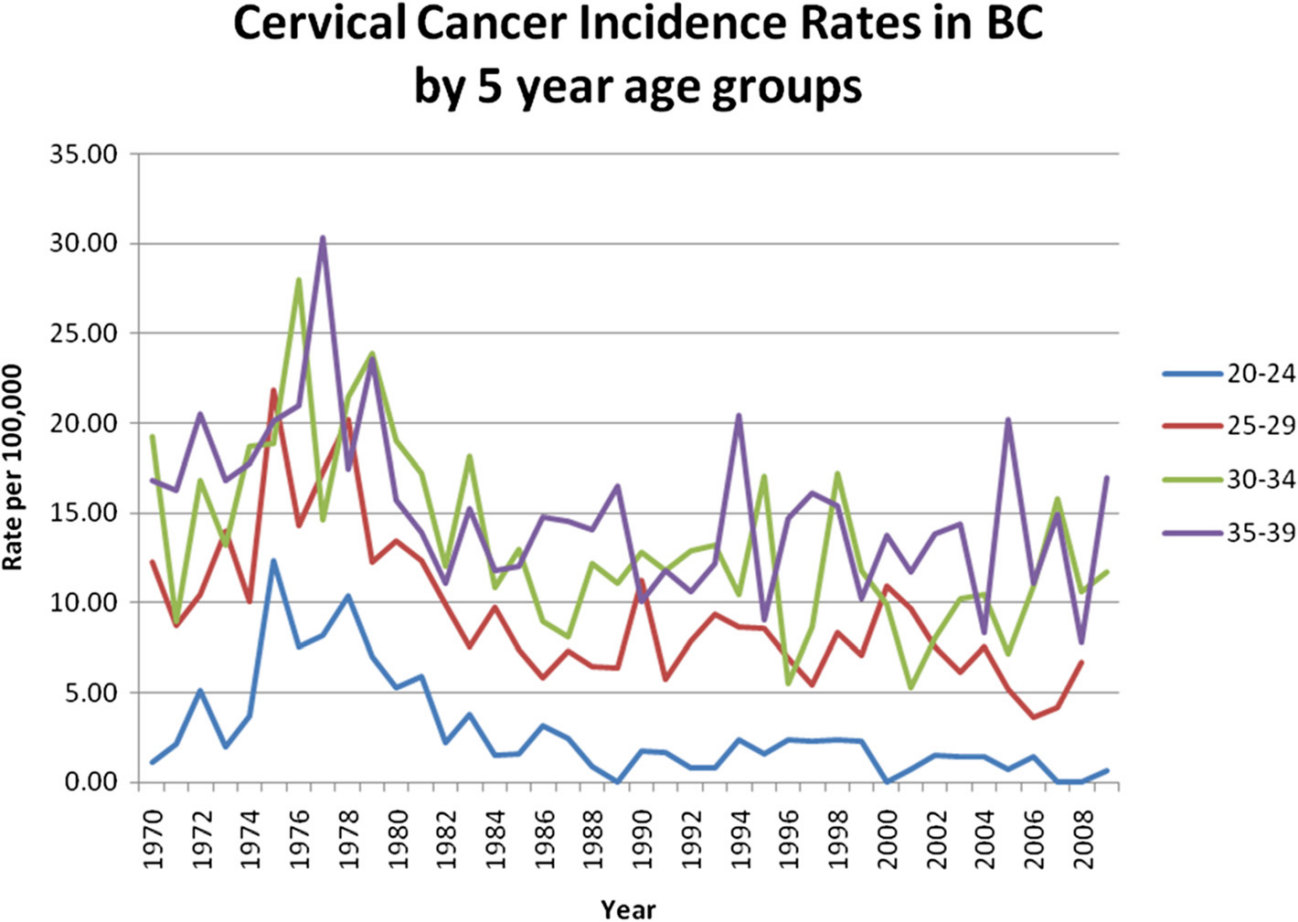
Cervical cancer rates in British Columbia, 1970–2008, by 5 year age groups, 20 to 39 years.

Reductions in both mortality and incidence are influenced by changes in the quality of death certification and disease registration, improvement in diagnosis and treatment, and screening for early disease. The increases in mortality before 1950 are likely due to classification errors (see data limitations). Subsequent reductions were initially probably related to introduction of effective treatment. In the post-World War II era of 1950 to 1970, the specialty of gynecology developed rapidly, initial treatment of cancer was done by well-trained gynecological surgeons, and radiotherapy became readily available across the country. Gynecological oncology as a specialty became widely available in the 1970s and 80s. These developments led to improved diagnosis and treatment, and likely contributed to the trends seen in mortality before 1975. Analysis of data from the Karolinska hospital in Sweden, together with trends in mortality from cancer of the cervix in that country, suggested that the introduction of radiotherapy for cancer of the cervix led to an important reduction in cervix cancer mortality [45]. Treatment techniques have only changed incrementally since then with addition of more effective chemotherapy [46], so are likely to have made smaller differences to mortality from about 1980, but mortality from cervical cancer in Canada continues to decline substantially. There was an apparent spike in incidence between about 1972 and 1980, most evident for women in the youngest age groups. This is clear in the data from British Columbia, a province that has a longer data series (Figure 8). Pathologists active at the time ascribed this to artefact due to the introduction of colposcopy, leading to more frequent use of punch biopsies that were difficult to assess, and produced more “over-diagnosis” of lesions that would regress. (personal communication D van Niekerk). This likely occurred across the country and exaggerated the apparent reductions in incidence based on national data from 1972–1976, especially at younger ages, where this artefact is greater in proportion to the total.

Screening rates in Canada have been consistently high from early ages and persisting until age 70, especially notable after 1996–7 when hysterectomy corrections were applied to the analyses (Table 3[47] These high proportions are not adjusted for sexual activity but screening has been associated with the practice of prescribing contraception and pre and post-natal care, therefore it is likely that most sexually active women are screened. This has occurred despite most screening being opportunistic: programs of systematic population-based reminders and recall have only commenced in recent years in some provinces [47]. Recent immigrants, those with lower incomes and rural women are less likely to have regular screening [48,49], so there is scope for improvement, as has been shown for aboriginal women [50].

A further factor that could affect both incidence and mortality of cervical cancer is change in the underlying epidemiology of infection and carcinogenesis by HPV. This could be influenced by behavior change or by immigration from countries with high cervical cancer rates. Relevant behaviour change may include early commencement of sexual activity, increased number of partners (not only of women, but of their consorts), smoking, and oral contraceptive use [12]. These might be expected to increase incidence rates, especially from the late 1960s when oral contraceptive use became widespread, and women in the cohorts born between 1930 and 1950 steadily increased smoking behaviour. Further, Canada has experienced substantial immigration since the 1950s, initially from Europe, then from various Asian countries, South America and Africa. Many of these areas have high cervical cancer rates [1], but immigrant women are less likely to be screened [48,49], so immigration may have attenuated the decrease in incidence and mortality that is observed.

Cervical screening is effective largely because it increases detection of pre-cancer (CIN, Ca-in-situ), and treatment prevents progression to invasive cancer [16]. Therefore the observed reductions in *incidence* are most likely due to screening, especially since risk factors have increased. If cancer still develops in a screened population, it is usually detected at an earlier stage and is therefore more amenable to curative treatment, so the program reduces *mortality* by two mechanisms. Some effect of screening began from 1949, in parallel with improved treatments, and in the 1960s when screening became widespread, mortality dropped further and was clearly related to screening [11]. Since then it has dropped yet more, despite minimal advances in treatment and the increased risk factors noted, so the continuing reduction can be ascribed to screening. From the 1990s, the rate of decrease has flattened out: which may be partly due to greater difficulty in reaching some segments of the population, and partly from reaching the limit of what can be achieved with screening. Adenocarcinoma of the cervix is less amenable to cytological screening, and while this used to comprise a small fraction, as squamous carcinomas are removed, they become a larger fraction [51,52]. In addition, more rapidly growing cancers are difficult to detect by screening and to treat [16].

Countries that have not instituted widespread screening have observed little change in cervical cancer mortality or incidence [53]. Substantial drops in incidence and mortality from cervical cancer in Canada have followed a different pattern from those found in the United Kingdom (UK) and some other European countries over the same period [17]. Canada showed a substantial reduction of incidence about a decade before the UK, where gynecological oncology treatment was also well developed but a major program of organized cervical screening was not introduced until 1988 [16]. The United Kingdom has observed fluctuations of cervical cancer incidence that relate to behaviours: women who reached early adulthood in times of war had higher rates than those before and after [14,15]. By contrast, Canada experienced no increase that can be ascribed to infection during the war years, nor after the introduction of oral contraception in the late 1960s.

The effect of screening is age dependent: mortality changes in the age groups under 25 are too small to assess in this analysis, and for those aged 25–29, most of the reduction occurred before 1972, so may have been as much due to treatment as screening. The greatest reductions both in proportion and numbers occur in mature women, about half before 1972–6. Thus it is likely that treatment reduced mortality, but screening added to this effect. Since it became widespread, screening has probably reduced the need for treatment of advanced disease, but at the cost of treating minor changes that are diagnosed as possible malignant precursors, causing artefactual increases in incidence especially among young women at the time that colposcopy was introduced. This artefact makes it difficult to accurately quantify the effect of screening on incidence among the lowest age groups. A limited effect of screening on reducing incidence under the age of 30 was found in a UK case control study [54].

The reduction in mortality from cervical cancer has been very similar in Canada, the United States and Finland [16]. Finland has an organized program, with screening every 5 years, largely restricted to women age 30–59. Programs of annual screening in North America over a much wider age range than Finland have placed a greater burden on women, causing more harm in terms of follow-up and over-treatment of early disease, and used far more medical resources, especially among women under age 30 years [13]. An IARC working group has recommended that screening should not start in any country before the age of 25 [12]. As the era of HPV testing arrives, policies must be planned to obtain a better balance of benefit, effort and harm, across all ages.

### Limitations of the data

This analysis was limited by four issues: misclassification prior to 1950, adjustment for hysterectomy, small numbers of cases among young women, and data availability. Firstly, although there have been no substantial changes in classification of cervical cancer in the ICD codes, prior to the 1970s there was misclassification of advanced disease between cervical and uterine cancer [6]. Consequently, early mortality data is likely to underestimate cervical cancer deaths. Much of the apparent rise in mortality prior to 1950 is probably due to improved classification. Secondly, incidence and mortality rates were not adjusted for hysterectomy and are therefore underestimated. This is particularly relevant for estimates among women over 45 years of age, when most hysterectomies occur. In Canada the hysterectomy rate peaked in approximately 1972, and prevalence was as high as 30% for women over 55 years [55]. However, there is insufficient hysterectomy data for us to accurately estimate hysterectomy-corrected rates by age over the whole period. An analysis on data to 1976 suggested that changing hysterectomy rates did not affect the fall in cervical cancer due to screening [56]. Since then, declining hysterectomy rates may have increased the denominator of women with an intact uterus and masked some of the subsequent effect of cervical screening. Thirdly, small numbers of cases and deaths in the youngest age groups make the rates susceptible to spurious variation related to small changes: including the effect that we identified of introducing colposcopy in the 1970s, affecting especially women under 30 years. Lastly, it is desirable to analyze the incidence and mortality reductions in relation to variations in provincial screening policies, availability and uptake of screening, and changes in treatment. Sufficient data is not available to enable this, and differences between provinces are relatively small in recent years [47].

## Conclusions

In Canada, high mortality from cervical cancer in the 1950s has changed to rates that are among the lowest in the world. The lifetime risk of being diagnosed with cervical cancer has fallen from 1.5% to 0.66% and the risk of dying has fallen from 0.92% to 0.22%. These effects are remarkable, given that about 20% of women do not get smears, or have them infrequently [47]. Many of the invasive cancers occur among these women, who ultimately may present with late stage disease. Thus the protective effect for those who have regular cervical smears is high. Despite more sporadic screening, most of the disease reduction has occurred among women over 40 years. Although initial improvements in mortality were likely related to improved treatment more recent improvements are probably largely attributable to the effect of screening, which likely has also reduced the need for more aggressive treatment.

Among women below the age of 30 years, cervical cancer rates have always been low, and have changed little, so it will be difficult to discern any effect of HPV immunization until the immunized cohort reaches beyond the age of 30. The approach to cervical screening policy may need to change in order to obtain the full benefit possible. Rather than starting screening at young ages and frequent re-screening of those who already participate, further advances are likely to arise from focusing on the sub-groups of the population that still have low screening rates.

## Competing interests

The authors declare that they have no competing interests.

## Authors’ contributions

The paper topic was originated by JD, with assistance from JO and CP in developing the questions and analysis outline. Data analysis was conducted by AS and LP. The paper outline was written by JD, whereas CP, LP, AS, AM and JO edited the paper. The final paper version was approved by all authors.

## Pre-publication history

The pre-publication history for this paper can be accessed here:

http://www.biomedcentral.com/1471-2458/12/992/prepub
